# Decentering America: the Quest for a Transnational American Studies

**DOI:** 10.1007/s12115-018-0285-3

**Published:** 2018-07-30

**Authors:** Rob Kroes

**Affiliations:** Amsterdam, The Netherlands

**Keywords:** Exceptionalism, Transnationalism, American studies, Phenomenology, Intertextuality, Historical palimpsests, Ideology

## Abstract

The planned removal of a Civil War monument in Charlottesville, Virginia, was the pretext for a white supremacist rally there in August 2017. It brought American fascists back into the streets, marching under the banner of a virulent nativism, of a vicious fear of being removed from the pedestal of their proper place in society. It also brought to the minds of people watching these images on TV older visual repertoires dating back to Nazi-Germany, fascist Italy, and similar racist clashes elsewhere. In such a stream of consciousness, such a chain of visual recollections, national settings—American or otherwise—are transcended. The wandering—and wondering—mind of the observer moves in a space naturally trans-national. The following essay considers the implications of such mental processes for the established forms of discourse among historians.

What came to my mind as a possible angle for the following discussion of transnationalism is based on an epiphany that I experienced while writing the last chapter of my recent book— *Prison Area, Independence Valley: American Paradoxes in Political Life and Popular Culture* (2015). The chapter revisits the concept of exceptionalism and argues on behalf of a version of methodological transnationalism. It does so by retracing the process that had unconsciously guided my hand when I wrote the book, a process that one might call mental intertextuality. Rereading my text, I noticed that whatever the precise topic, be it the history of the freak show and public spectacle, or the trajectory of atrocity photographs, such as Holocaust images, in my mind one image under discussion evoked related images, thematically related yet originating in different geographical and historical settings. Thus, through mental intertextuality, an argument could evolve that naturally transcended geographic, historical, and cultural borders, freely ranging in a transatlantic space. The outcome was unintentional, yet undeniably transnational.

My discussion of American exceptionalism brings out this process more clearly and presents it as a natural counter-trajectory, highlighting associative processes in our minds as inherently transgressive, circling back and forth and constantly affecting our reading of whatever is before our mental eyes. It moreover affects our reading of the concept of American exceptionalism. Letting ourselves be guided by the flow of mental intertexts, an alleged American exceptionalism gives way to transnationalism, in much the same way that in palimpsests surface texts and images cover what went before, yet never quite erasing what lies submerged beneath them.

The following argument will highlight selected moments illustrating my confrontation with such palimpsests, scratching away the surface to find myself literally transported from one historical setting to another, confronting historical parallels, or better: a circulation of ideas and images across the Atlantic, transcending national settings and contexts.

In a nationally televised speech on Syria, September 10, 2013, President Obama turned to American exceptionalism as a rallying cry in his endeavor to unite his country behind him. “America is not the world’s policeman. Terrible things happen across the globe, and it is beyond our means to right every wrong,” Obama said. “But when, with modest effort and risk, we can stop children from being gassed to death, and thereby make our own children safer over the long run, I believe we should act.” These are words one might expect from a political pragmatist; they are far removed from the ringing global pitch of, say, Roosevelt’s Four Freedoms. Yet, as if eager to take a leaf from Rooseveltian rhetoric, Obama went on to say: “That’s what makes America different. That’s what makes us exceptional.” The concluding word must have brought a wry smile to some at least among his listeners. They must have recognized its use not as powerful rhetoric, but rather as formulaic, as a shibboleth granting safe passage to a man whose political credentials had never been fully accepted by a vengeful part of the American citizenry. The word *exceptional* had become the shibboleth to those in the media and the political arena who were out to de-construct and undermine the president from the moment he had entered office.

Obama may have quickly learned his lesson, paying tribute, if not lip service, to a word that was of relatively recent currency in American political discourse. The role it played, though, was similar to that of earlier passwords like Americanism and anti-Communism, as in the days of the Red Scare following World War I or in the early years of the Cold War with McCarthyism in the role of monitor and protector of the purity of the body politic. The monitoring gaze today comes once again from the political right, embodied in its lunatic fringe of the Tea Party and the Alt-Right.

Yet it would be wrong to see Obama as merely paying lip service to the word exceptionalism and all it stands for in summary of a larger American creed. Many have been the occasions, from his early presidency on, where we can see Obama revisiting the concept, not just to pay tribute and be done with it, but to consider the options it gave him to be an educator of the nation, to bring a degree of complexity to a word that too often was used as a facile trope. The way Obama used the word was very much in the vein of what Sacvan Bercovitch called the American Jeremiad, a specific form of public speech that reminds the audience of its high calling while pointing to the many ways in which it is still falling short. Listen to Obama, in 2008:

We have a core set of values that are enshrined in our Constitution, in our body of law, in our democratic practices, in our belief in free speech and equality, that, though imperfect, are exceptional.

Now, the fact that I am very proud of my country and I think that we’ve got a whole lot to offer the world does not lessen my interest in recognizing the value and wonderful qualities of other countries, or recognizing that we’re not always going to be right, or that other people may have good ideas, or that in order for us to work collectively, all parties have to compromise and that includes us.

I see no contradiction between believing that America has a continued extraordinary role in leading the world towards peace and prosperity and recognizing that leadership is incumbent, depends on, our ability to create partnerships because we can't solve these problems alone. (Obama qtd. in “The Big Lie”)

In the eyes of the right, qualifying words like “though imperfect,” or the call for compromise, while acknowledging that other people may have “good ideas,” may already be far too subtle. But what caused them to rise in howling anger were Obama’s opening words— often the only words quoted in the right’s indictment: “I believe in American exceptionalism, just as I suspect that Brits believe in British exceptionalism and the Greeks believe in Greek exceptionalism.” As right-wing commentator Michael Barone thundered: “One cannot imagine Presidents Roosevelt, Truman or Kennedy, Eisenhower or Reagan, uttering such sentiments” (Barone qtd. in “The Big Lie”).

Up against such odds, a man like Obama, politician *and* intellectual, must tack to political winds while keeping an eye on the compass of his convictions. He has kept valiantly trying to add a touch of realism and relativism to the idea of American exceptionalism, much as that very endeavor is an abomination in the eyes of the Tea Party watch dogs. To those with a historian’s memory, however, it may even appear as if Obama was trying to add an almost European sense of the fallibility and frailty of human exploits to counter the more impetuous uses of exceptionalism in American political discourse. I for one could not help being reminded of C. Vann Woodward’s reading of the historical experience of the American post-Civil War South as the only region in the United States to have experienced defeat and loss and to have developed a quasi-European sense of the tragic. Some of that sobering sense, I feel, is what Obama was struggling to convey to a larger American public.

There is a further irony here. If my reading of the gist of Obama’s revisits of the concept of exceptionalism is correct, it would highlight a resemblance between his aims and current trends in the academic study of the United States. What C. Vann Woodward in his day had still to call a “counterpoint” to the prevailing mainstream reading of American history has now become a widespread inspiration in the fields of American history and American Studies. The urge began to be felt from the 1990s onwards to break out of a conceptual view of America as *sui generis*, as exceptional, as different in its historical experience and destiny than any other country or nation in the world. Surely, the sense of American difference had been around for much longer and had in fact inspired explorations of the many ways in which America had proved different than other countries, though not exceptional. The best encyclopedic treatment is Seymour Martin Lipset’s *American Exceptionalism*.

A comparativist, Lipset looked at areas in political and social life where America traditionally was seen as forming an exception to rules prevalent in Europe. Thus, he revisited Tocqueville’s *aperçus* concerning the lasting effects of America’s special historical genesis and development, and the German early twentieth-century historian and sociologist Werner Sombart’s classic study on the question of why there is no socialism in the United States. They are all areas where America can be seen to offer counterpoints to European history while in other areas it moved in step with European history. Thus, America could be woven into a larger narrative of forces of social change and modernization as these affected nations on both sides of the Atlantic, each with its own peculiar quirks and twists. Yet exceptionalism—in its more demanding, exclusivist reading—is a different animal. It has taken more than a little pushing to shatter its hold on American historiography and on the American sense of identity.

In an influential essay entitled “Exceptionalism,” Daniel Rodgers made the point that from the early modern era to the postcolonial present, the cultivation of sentiments of difference and superiority has been at the heart of the project of nation-state formation. Within these common terms, however, there has run a thread which, if not wholly distinct to the American complex, holds a peculiar prominence there. That is the idea of exceptionalism. Rodgers then makes the following simple, but crucial point: Exceptionalism differs from difference. Difference requires contrast; exceptionalism requires a rule. Exceptionalist claims pin one’s own nation’s distinctiveness to every other people’s sameness—to general laws and conditions governing everything but the special case at hand. When difference is put in exceptionalist terms, the exception becomes an exemption, an exemption from the universal tendencies of history, the “normal” fate of nations, the laws of historical mechanics itself (Rodgers, *Atlantic Crossings*).

It is implications like these, where a nation can claim to be above the general rule, if not above the law, that have inspired America’s political action as much as its self-reflection. If other nations have agreed to set up an International Criminal Court, America is no party to it, refusing to abide by rules that others have subjected themselves to. Yet among American academics strong movements have occurred to do away with exceptionalism in their understanding of the driving forces behind American history. Programs aiming at “transnationalizing” or globalizing the intellectual paradigms of American history and American Studies found wide support in the main professional organizations. Daniel Rodgers published a pioneering study, entitled *Atlantic Crossings*, that illustrated the gains to be had from internationalizing the frame of interpretation. *Atlantic Crossings* is the first major account of the vibrant international networks that American reformers, Progressives and, later, New Dealers constructed and of its profound impact on the United States from the 1870s through 1945, a story so often obscured by notions of American exceptionalism. At about the same time two collections of essays were published with the broad support of the Organization of American Historians (OAH), edited respectively by David Thelen and Thomas Bender. Both publishing projects broadly aimed at questioning the nation-centered focus of American history, as Thelen has it, or as Bender puts it: “To historicize the nation is to relate its dominant narrative, its national narrative, to other narratives that refer to both smaller histories and larger ones. That means understanding the historical production of the nation and locating it in a context larger than itself” (vii).

Words like “the historical production of the nation” betray an affinity and intellectual exchange with yet another community of students of America, those active in the intellectual domain of a self-styled American Studies. Always more open than their colleagues in academic history departments to intellectual perspectives current among cultural studies scholars, more willing to use a language that emphasizes the constructionist elements of reality, of reality as imagined and collectively formed through social interactions, American Studies people had begun to move in new directions following what was commonly referred to as “the cultural turn.” It was just one of the fashionable turns they had collectively taken in recent years, turns such as the linguistic turn, the visual turn, the transnational turn. It had left some wondering how many turns it takes before the wheel is reinvented. For indeed, what seemed like a paradigm shift under the banner of the cultural turn, from a larger intellectual perspective may well be seen not as a new turn, but as a return to the wisdoms of the old Chicago School in the social sciences, with its seminal insights into social reality as the product of collective construction by all participants.

Whatever the case, adherents of the New American Studies set upon the “de-construction” of their own academic field with a vengeance. At times their efforts showed a vehemence as if the issue was a matter of exorcism, of driving out all the evil connotations of the word “America,” in an act of linguistic voluntarism, as if changing the language one used would change the world. It led one outsider to scathingly speak of Anti-American Studies, in a facetious review in the *New Republic* of three examples of the new post-exceptionalist American Studies (Wolfe).^1^

It was not long, though, before sobering second thoughts came to some of the leading “New Americanists.” In a piece entitled “Re-thinking ‘American Studies after U.S. Exceptionalism’,” Donald Pease acknowledged the resistance to change of large swathes of reality. “Transnational American Studies aspired to remediate the discourse of U.S. exceptionalism by transnationalizing the core values of American civil society. But global civil society has neither transcended the era of the nation-state nor entered into the utopian realm of a cosmopolitan democracy. Have not scholars in transnational American Studies,” he asked, “overestimated the ways in which global civil society can mobilize the political energies needed to remedy the economic inequalities that globalization has engendered? Has not post-exceptionalist American Studies also ignored the U.S. state’s power to describe the US as a permanent state of exception?” (22). Strange things are happening in this one paragraph. Not only does it describe the changes sought by the transnational turn in American Studies as changes in language, as if these would be enough to change the world. It betrays an attitude that I choose to call linguistic voluntarism. At the same time, the paragraph describes the intrusion of what Freud would have called the reality principle, of the hard facts of economic inequality and the permanent state of military mobilization as a quasi-enduring ‘state of exception.’ There is a remarkable return here, linked undoubtedly to the aftermath of 9/11 and the American display of what is known among military people as “full spectrum dominance,” to age-old concepts like the state and the state’s power, or for that matter the nation-state and its attendant nationalism. The permanent state of exception, in an ironic pun, is presented here as a product of the state’s power, as the outcome of the state’s power to manipulate reality for its citizenry, through such language as ‘the war on terrorism,’ or the threat of Jihadism. We mentioned linguistic voluntarism before, but if one needs proof of it happening, here it is, as used by the powers-that-be. 000.

Clearly the work of “re-mapping the transnational”—the name of the series in which my book came out—is a work in progress, a long-term project. How do I see the place of my book in the larger project? For one thing, for much of my life as an academic active in American Studies at the University of Amsterdam, one continuing theme has been my study of the many ways in which American and European cultures have cross-pollinated and the ways in which cultural influences were received or resisted. Part of my interest was in issues of Americanization of European cultures or of European anti-Americanism, on either political or cultural grounds. Some chapters in my book clearly reflect that interest, while also critically revisiting it. If issues of empire and imperial sway show up in my writing there, it is clearly in response to wider intellectual concerns in the post-9/11 study of America. Issues of politics and power have forced themselves upon my mind most directly in the opening and concluding chapters of the book, on the George W. Bush administration first, on the Obama administration later. The chapters were written against the backdrop of general mood-swings, both in the U.S. and in Europe. There is one more general aspect of transnationalism, though, that I only became aware of while writing the book. In my earlier writing on American popular culture, I tried to answer questions as to what accounts for the lure and appeal of American popular culture, at home and abroad. In my latest book, though, I found that my interest had moved to the darker side of popular culture and public spectacle, even in such extreme varieties as lynchings.

I also found, more clearly than ever before, that there are forms of trans-nationalism inherent to the train of thought of the human mind. Addressing spectacles and parades as forms of public entertainment, I noticed my mind wandering from circus and side-show artists parading through American small town Main Streets, to dignified Jewish citizens being forcibly paraded through German cities on the day following Kristallnacht (the night of the shattered glass) to the merriment of German onlookers, and back from there to the many photographs of public lynchings in the American South, with jolly and grimacing bystanders posing for the camera. The most notorious among this latter corpus are photographs to do with the Ku Klux Klan. After a long spell of quiescence, it reemerged into national prominence in the 1920s, reaching an all-time peak membership in 1924—a year, incidentally, that saw the dedication of various Confederate memorials, including the Robert E. Lee statue in Charlottesville, Virginia. It was its planned removal that served as pretext for the “Unite the Right” —also: “They will not replace us”—rally there, in August 2017. Stunningly, in our present day and age, it brought American fascists back in the streets, marching under the banner of a virulent nativism, of a vicious fear of being removed from the pedestal of their proper place in society. It also brought to the minds of people watching these images on TV older visual repertoires dating back to Nazi Germany, fascist Italy, and similar racist clashes elsewhere. In such a stream of consciousness, such a chain of visual recollections, national settings—American or otherwise—are transcended. The wandering—and wondering—mind of the observer moves in a space naturally trans-national.

I thus found, more clearly than ever before, that there are forms of transnationalism inherent to the workings of the human mind. I became increasingly aware of my own thought processes while putting its results down on paper, in other words: my thinking reflected back on itself. If there is a methodology here, it is one that may remind us of *phenomenology*, as introduced by German philosophers like Edmund Husserl in the late nineteenth century. Thus, in my case, writing about freaks in 1930s America—some of whom had been immigrants from Germany—writing about the Lilliput town on Long Island that housed them, styled after the German medieval city of Nürnberg, brought images to my mind of Nazi Germany, its persecution of freaks, and its Nürnberg race laws. Images of the Nazi holocaust in their turn called up pictures in my mind of atrocity photographs as they had circulated in the United States after the war.

Similarly, in a piece about anthropological shows or human zoos as they were also called, immensely popular in the late nineteenth century, my argument shifted to the way that our current tastes and sensibilities now forbid us to enjoy what was popular entertainment only a century ago. The central illustration in my argument was the Buffalo Bill Wild West show (Kasson). Buffalo Bill was the supreme master in almost instantly translating recent American frontier history into spectacular entertainment. Mark Twain, connoisseur of contemporary American idioms, praised Buffalo Bill, telling him on the eve of Buffalo Bill’s first European tour to England that he could show Europeans something that was authentically American. Twain chose to ignore that Buffalo Bill’s Wild West show presented a sanitized version of the American West, leaving out any reference to recent tragedies suffered by American Indians. This is the more remarkable coming from a man who was a leading voice in the international campaign protesting atrocities perpetrated in the Belgian Congo. There he could see through the self-serving lies of Belgian colonialism. Twain would have none of it; instead he wrote a biting indictment which he called “King Leopold’s soliloquy.” It showed real images of the atrocities inflicted on the native population, maimed and mutilated in the Belgian policy of colonial extortion. Again, putting such images alongside photographs, widely circulating, of native American suffering – showing Indians killed in the 1891 Wounded Knee massacre, frozen stiff in contorted poses - leaves one wondering how a man like Twain could have managed to live with both versions of reality at the same time: praising Buffalo Bill’s entertainment version, while closing his eyes to a history of atrocity and suffering that he so clearly saw and denounced in the Belgian case. To him there were no continuous visual associations connecting the two settings. To our modern post-colonial mind, it may be easier to move between repertoires of visual representation.

It is not only a matter, though—and I wish to emphasize this—of a process of visual associations forcing itself upon our minds. Following the same logic, the train of associations can also be linguistic, where an argument applying to one historical situation calls forth similar arguments applying to different situations. Thus, following my exploration of associated images of the American South and 1930s’ Nazi Germany, a book came to my mind—which I had read as a student and had been deeply impressed by—written by Kurt Baschwitz, a German Jew who had fled from Nazi Germany to the Netherlands. From his new refuge, he became aware of the historical parallels between mob behavior in Germany and the American South and the logic behind it. In what would become a classic study in mass psychology, published in exile in Amsterdam, the author saw his analysis of processes of mob behavior confirmed in both settings, in an amazing act of creating intellectual distance to current events even as they had such immediate dramatic relevance to his own life.

What I am trying to convey is that the transnationalism that one can see happening here, is almost like a chimera, with one image shimmering through another one, as if in a palimpsest. History does form palimpsests, covering one layer of images with later ones, as if on the wall of an old house with one painted advertisement not quite covering a preceding one. It is an uncanny experience when, by looking intently at one image, another one shows up in one’s mind, shimmering through, taking you from one locale and time to another. It is also an exhilarating experience, a sense of being literally transported in an exercise of transnationalism.

## Transnationalism as an Antidote to Exceptionalism?

Transnationalism, as here conceived, is a mental process as well as an intellectual exercise that we may use to resist the attractive force of exceptionalism. To give it that thrust, we need to take it away from its private, almost aesthetic, qualities. We can indeed enjoy these, intrigued by the flow of images as it runs before our inner eyes, following its own associative logic. But more is needed to turn it from a mere solipsistic act, not unlike the pleasure offered by the virtual worlds of a 3-D helmet, into an intellectual perspective. A measure of analytic control is needed to guide the associative flow and make it serve the point and purpose of an argument.

If the point is to confront transnationalism and exceptionalism, one obvious first step would be to zoom out from any specific instance of exceptionalism and to see it as just one case among many others. This is precisely what Obama did, conceiving of American exceptionalism as a specific case within the larger category—the larger *genus*—of national exceptionalisms. Hovering above the fray, in the manner of the true transnational mind, he showed American exceptionalism its place. The vehemence of the reaction to this perspective affirmed the rival reading of American exceptionalism as purely *sui generis*, as being one of a kind. This is what Daniel Rodgers made clear in his revisit of the concept of exceptionalism. In this extreme version, American exceptionalism comes to stand in logical opposition to transnationalism. In that version too, it turns from an analytic perspective into a national ideology, no longer open to disinterested discussion and intellectual debate. It becomes a password in the heated national debate setting insiders apart from outsiders. As such it is only the latest stage in a national pastime as old as the American nation, at whatever stage of its historical formation.

Transnationalism, then, appears as an unlikely contender for national self-reflection in the United States at its present stage. It is too relativistic, too ironic, to sustain and reflect the current national mood. For the time being it may well have to pull back inside the walls of academia. It may be a while before the United States finds a president as open-minded, as cosmopolitan, as the one who served two full terms in the White House, from 2008 to 2016.

## Footnotes

1 In his piece, Wolfe reviewed Donald Pease and Robyn Wiegman, eds., *The Futures of American Studies*, John Carlos Rowe, *The New American Studies* and David Noble, *Death of a Nation: American Culture and the End Exceptionalism*.
